# Ethnopharmacological Approaches for Therapy of Jaundice: Part I

**DOI:** 10.3389/fphar.2017.00518

**Published:** 2017-08-15

**Authors:** Devesh Tewari, Andrei Mocan, Emil D. Parvanov, Archana N. Sah, Seyed M. Nabavi, Lukasz Huminiecki, Zheng Feei Ma, Yeong Yeh Lee, Jarosław O. Horbańczuk, Atanas G. Atanasov

**Affiliations:** ^1^Department of Pharmaceutical Sciences, Faculty of Technology, Kumaun University Nainital, India; ^2^Department of Pharmaceutical Botany, “Iuliu Hatieganu” University of Medicine and Pharmacy Cluj-Napoca, Romania; ^3^ICHAT and Institute for Life Sciences, University of Agricultural Sciences and Veterinary Medicine of Cluj-Napoca Cluj-Napoca, Romania; ^4^Division BIOCEV, Institute of Molecular Genetics, Academy of Sciences of the Czech Republic Prague, Czechia; ^5^Applied Biotechnology Research Center, Baqiyatallah University of Medical Sciences Tehran, Iran; ^6^Institute of Genetics and Animal Breeding of the Polish Academy of Sciences Jastrzebiec, Poland; ^7^School of Medical Sciences, Universiti Sains Malaysia Kota Bharu, Malaysia; ^8^Department of Public Health, Xi'an Jiaotong-Liverpool University Suzhou, China; ^9^Department of Pharmacognosy, University of Vienna Vienna, Austria; ^10^Department of Vascular Biology and Thrombosis Research, Centre for Physiology and Pharmacology, Medical University of Vienna Vienna, Austria

**Keywords:** jaundice, bilirubin, medicinal plants, ethnopharmacology, traditional use, metalloporphyrin

## Abstract

Jaundice is a very common symptom especially in the developing countries. It is associated with several hepatic diseases which are still major causes of death. There are many different approaches to jaundice treatment and the growing number of ethnomedicinal studies shows the plant pharmacology as very promising direction. Many medicinal plants are used for the treatment of jaundice, however a comprehensive review on this subject has not been published. The use of medicinal plants in drug discovery is highly emphasized (based on their traditional and safe uses in different folk medicine systems from ancient times). Many sophisticated analytical techniques are emerging in the pharmaceutical field to validate and discover new biologically active chemical entities derived from plants. Here, we aim to classify and categorize medicinal plants relevant for the treatment of jaundice according to their origin, geographical location, and usage. Our search included various databases like Pubmed, ScienceDirect, Google Scholar. Keywords and phrases used for these searches included: “jaundice,” “hyperbilirubinemia,” “serum glutamate,” “bilirubin,” “Ayurveda.” The first part of the review focuses on the variety of medicinal plant used for the treatment of jaundice (a total of 207 medicinal plants). In the second part, possible mechanisms of action of biologically active secondary metabolites of plants from five families for jaundice treatment are discussed.

## Jaundice: an overview

**Figure 1:**
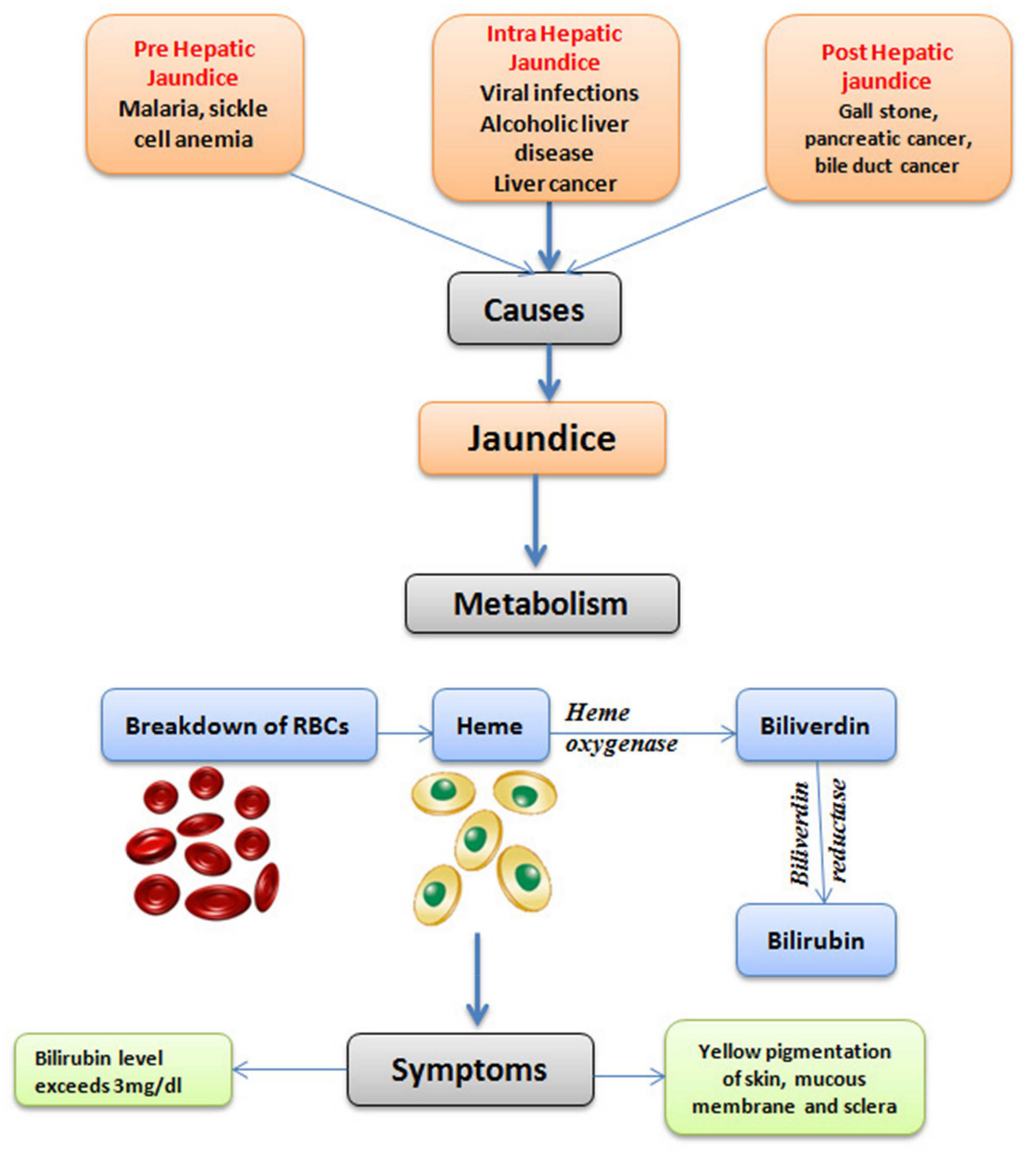
The overview of the symptoms and causes of jaundice and the metabolism of bilirubin.

Jaundice is one of the most wide spread disease conditions occurring throughout the world. It is also a life-threatening condition, mostly in the underdeveloped countries. Jaundice is caused by elevated serum bilirubin concentration in the body (Ullah et al., 2016). The term “jaundice” is derived from the French word “jaune,” which literally means yellow (Constantin, 2011). The metabolism of bilirubin takes place through the hemolysis of red blood cells (RBCs), which leads to the release of hemoglobin. The heme oxygenase degrades heme into biliverdin and carbon monoxide within the reticuloendothelial system. Biliverdin is then converted to unconjugated bilirubin by biliverdin reductase (Figure 1). The unconjugated bilirubin binds to albumin and is transported to the liver. The unbound unconjugated bilirubin is toxic to the central nervous system as it can cross the blood-brain barrier (BBB) (Brites, 2012; Olds and Oghalai, 2015; Jasprova et al., 2016).

**Figure 2:**
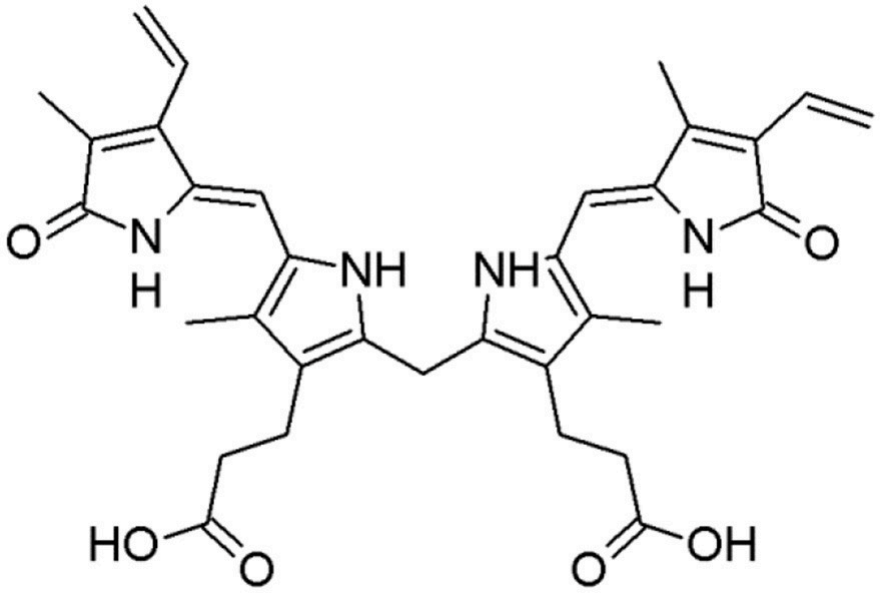
Structure of bilirubin.

Jaundice is mainly observed in neonates and is not common in adults. However, when it is present in adults, it suggests a serious predicament. In developing countries in particular, jaundice is very prevalent and can be life-threatening. The pathophysiology of jaundice and the metabolism of bilirubin take place in three phases: viz. pre-hepatic phase, the intra-hepatic phase, and the post-hepatic phase. A problem in any of these phases can lead to jaundice. Bilirubin is the metabolic (or breakdown) product of hemoglobin in erythrocytes (Figure 2). The heme metabolism has a central role for bilirubin production (Memon et al., 2016). Heme is an iron-containing porphyrin that is present in hemoglobin, myoglobin, and numerous enzymes, such as hepatic cytochromes (de Visser and Stillman, 2016). It is estimated that 80% of bilirubin formation takes place through heme breakdown in reticuloendothelial cells, spleen, or liver.

The unconjugated bilirubin is largely insoluble in water, but can be reversibly conjugated to albumin. It is transported to the liver, escaping the filtration in kidneys. Generally 90–95% of the bilirubin circulating in the blood is unconjugated. In the case of hypoalbuminemia (a type of hypoproteinemia), bilirubin displacement from the albumin molecules may cause diffusion of bilirubin across the BBB. This is by means of an assortment of drugs and/or increase in the blood unconjugated bilirubin levels. If the higher amount of bilirubin crosses the BBB and the level of unconjugated bilirubin in the blood reaches 15–20 mg/dL, it causes bilirubin encephalopathy, or kernicterus (a bilirubin induced brain dysfunction; Kruger, 2011). The conjugation of bilirubin takes place across the sinusoidal membrane within the hepatocytes after the hepatic uptake, followed by the action of microsomal uridine diphosphoglucuronyl transferase (UDPGT), converting bilirubin to water-soluble form and facilitating its excretion into bile and by the urine (Kruger, 2011). The metabolism of heme results in about 4 mg/kg/day bilirubin production. Maximum amount (about 80%) of heme moiety is utilized by the catabolism of erythrocytes and the rest 20% resulting from the ineffective erythropoiesis and breakdown of muscle myoglobin and cytochromes (Pashankar and Schreiber, 2001).

The major manifestation of jaundice is the yellow color of skin and mucous membranes due to deposition/gathering of bile pigments in blood and body tissues by bilirubin. The color expression is the same in case of carotenemia, but in this condition the bilirubin levels are normal (Kruger, 2011; Schlosser et al., 2011). The pigment depositions are of little effect for most newborns, but in preterm infants even lower doses of bilirubin have the potential for causing kernicterus (Maisels, 2000; Santos et al., 2008). Adults usually have a normal range of serum total bilirubin concentration <1 mg/dL (Roy-Chowdhury and Roy-Chowdhury, 2011). There are several parameters by which the jaundice can be diagnosed once the bilirubin concentration reaches 2.0–2.5 mg/dL. However, even experienced clinicians may not observe the yellow skin coloration until the serum bilirubin levels reach 7–8 mg/dL. It is believed that this coloration is clinically detected once the serum bilirubin level increases above 3 mg/dL (51.3 μmol/L; Roche and Kobos, 2004).

The occurrence of jaundice (icterus neonatorum) in newborns is observed for centuries. Juncker and Stahl (1724), in the *Conspectus Medicinae Theoretico praticae* distinguished “true jaundice” and “the icteric tinge which may be observed in infants, immediately after birth” (Lauer and Spector, 2011). A subcommittee on hyperbilirubinaemia estimated that apparent jaundice developed approximately in 60% of full term babies along with 85% of pre-term babies and about 10% of breast fed babies are still jaundiced at age of 1 month (Dennery et al., 2001; Zeitoun et al., 2013). The neonatal jaundice is apparent on day 3, peaking on day 5–7 and resolving by day 14 is called “physiological jaundice,” and it is considered as normal physiological condition. The “physiological” jaundice is usually benign, however brain injury and lifelong disability can occur if serum bilirubin (unconjugated), which is neurotoxic, is too high level and crosses the BBB causing damage of auditory nerve and basal ganglia (Subcommittee on Hyperbilirubinemia American Academy of Pediatrics, 2004). Such condition is rare (about seven new cases each year in the United Kingdom; Manning et al., 2007) and sequel include deafness, choreoathetoid cerebral palsy, and upgaze palsy. The signs and symptoms of jaundice are associated with serious liver disease, such as biliary atresia, whose treatment should be done at the age of 6 weeks (Hartley et al., 2009; Lauer and Spector, 2011). It should be noted that the disease imposes large social costs (Ebrahimimd et al., 2011). Most commonly accepted treatment of hyperbilirubinemia is phototherapy. However, there are several complications like dehydration, retinal injury, bronze baby syndrome and diarrhea, which can put the baby at risk (Stoll and Kliegman, 2004; Kliegman et al., 2007; Ebrahimimd et al., 2011).

## History of jaundice

The history of jaundice is very long and is described as a sign of “causeless hatred” in the Babylonian Talmud (Poduri, 2016). There are various ancient references related to jaundice which are presented in Babylonian Talmud, Sumerian Tablets, Ebers papyrus, and in Ancient Ayurveda (the Indian traditional system of medicine; Poduri, 2016).

Moreover, the work of Hippocrates (460–370 B.C.) also provided references to jaundice (Schmid, 2001; Bynum, 2008). The terms that are used in hepatology like “hepatic,” “liver,” and “jaundice” have been originated from Greek, Sanskrit and French languages, respectively (Riva et al., 2011; Definition, 2016)^1^. The most primitive Latin word used to indicate the liver was “iecur,” which is likely from Sanskrit “*yakrt*” (Riva et al., 2011). The word “jaundice” derives from an old French word “jalnice,” followed by “jaunice,” which means “yellowness.” In earlier times before 1800, there was limited notion about jaundice. Its relation to the alcohol uptake was described in 1836 by Addison (Gao and Bataller, 2011). In 1885 Lühraman recorded the occurrence of jaundice as an adverse effect of vaccination (Thomas et al., 2013; Trepo, 2014). In 1908 McDonald suggested that the probable cause of jaundice might be an agent, which was much smaller than bacterium (McDonald, 1908; Thomas et al., 2015). This idea was developed in 1923 by the hypothesis that virus was the cause for jaundice (Thomas et al., 2013; Poduri, 2016). A great number of death cases were observed during World War II (WWII) by hepatitis (1939–1945). During WWII, it was estimated that around 16 million people were killed as a consequence of hepatitis (Trepo, 2014). Therefore, different liver disease conditions like autoimmune hepatitis, liver cirrhosis, hepatitis A, B, C, D, E, hepatic carcinoma, or hemolytic anemia may lead to jaundice (Wahab et al., 2004; Amiri et al., 2014).

## Discovery of hepatitis viruses

Most common hepatitis viruses are categorized as hepatitis-A (HAV), hepatitis-B (HBV), hepatitis-C (HCV), hepatitis-D (HDV), and hepatitis-E (HEV). Although, HBV, HCV, and HDV are spread mainly by blood exposure, HBV is transmitted mostly by unprotected sex. On the other hand HAV and HEV are spread predominantly by fecal-oral means (Singhal et al., 2009; Puri, 2014). A brief account on the discovery of these hepatitis viruses are presented here:

### Hepatitis A virus

HAV belongs to Picornaviridae family of the genus Hepatovirus. Seven genotypes of HAV in the feces may be seen and excretion of 106–1,011 viruses per gram of feces was recorded for HAV (Coudray-Meunier et al., 2014; Sánchez, 2015; Bennett et al., 2016). The origin of HAV may be traced back to epidemics of “campaign jaundice,” which afflicted the armies of Middle Ages and it has been a serious problem during the Korean and Vietnamese conflicts (Zuckerman, 1976; Beaumier et al., 2013). Every year around 1.4 million new cases of HAV infection are reported around the world [http://www.who.int/csr/disease/hepatitis/whocdscsredc2007/en/index4.html (Accessed on July 13, 2016) Kim et al., 2017].

### Hepatitis B virus

HBV belongs to the family Hepadnaviridae and genus Orthohepadnavirus (Tajiri and Shimizu, 2016). HBV isolates are categorized into 10 groups of genotypes with nucleotide sequence divergence of 7.5% or higher (Tong and Revill, 2016; Zhang et al., 2017). It is an enveloped DNA virus with presence of transcriptional template [3.2-kb covalently closed circular (ccc) DNA] in the infected hepatocytes nucleus (Seeger and Mason, 2015; Zhang et al., 2017). Lurman (1885) reported the occurrence of an epidemic of hepatitis in the Bremen's Shipyard workers, following vaccination against smallpox with glycerinated lymph of human origin in 1883. In 1970 transmission of hepatitis B was first carried out in chimpanzees. In 1981, the first vaccine against hepatitis B was licensed in the USA, which was prepared by purified hepatitis B surface antigen (HBsAg), a viral envelop protein from the plasma of chronically affected individuals (Geier et al., 2003).

### Hepatitis C virus

HCV belongs to the genus Hepacivirus which is a hepatotropic enveloped, positive-stranded RNA virus of Flaviviridae family (Elgharably et al., 2017). Seven major and more than 12 minor genotypes of HCV have been identified based on the genomic sequence heterogeneity (Petruzziello et al., 2016). Until 1975, hepatitis A and B were recognized since the diagnostic tests were available for both. However, hepatitis C was reported as a non-A non-B virus from stored samples of transfusion associated with hepatitis (Feinstone et al., 1975; Kim et al., 2016). The viral RNA was identified in 1989 by reversed-transcription PCR, and the subsequent cloning and sequencing led to the description of the whole genome (Kuo et al., 1989). Only few proteins out of the entire viral protein pool are serving as a base for different serologic tests and anti-HCV antibodies.

### Hepatitis D virus

Existence of HDV was found by immunofluorescence in liver biopsies from Italian patients with chronic HBV infection in 1978 as previously uncharacterized intranuclear antigen. The antigen is coupled with the capsid protein of previously unrecognized virus, afterwards termed as “hepatitis delta virus” (HDV; Rizzetto and Verme, 1985; Taylor, 2006). HDV was distinguished and did not share any homology to the other known animal viruses and resembled the plant virus satellites, which are also related to plant viroids (Sureau and Negro, 2016). The HDV genome consists of single-stranded RNA and eight genotypes have been identified from different geographical regions (Opaleye et al., 2016).

### Hepatitis E virus

HEV, an RNA-containing virus which is the only virus of genus Hepevirus belonging to family Hepeviridae. The virus is classified in four genotypes distributed all over the world (Fierro et al., 2016). Hepatitis E was recognized as a unique human disease in 1980 by examining stored clinical samples collected during water-borne epidemics of viral hepatitis A and B in Indians (Khuroo, 1980; Wong et al., 1980; Fierro et al., 2016). The massive epidemic of hepatitis, which occurred in Delhi, India during 1955–1956 by contamination of a major water treatment plant with raw sewage, was considered as a classical example of water borne hepatitis A (Kumar et al., 2013). The consequent studies revealed that the Indian population is highly endemic to hepatitis A, as HAV infected almost 100% of the population by the age of 5–10 years, which made it difficult to discriminate HAV from other causes before Delhi epidemic and other water born epidemics that occurred in young adults. In 1983 HEV was transmitted to a human volunteer and cynomolgus monkeys and visualized for a first time and its role in enteric transmitted NANB (ET-NANB) hepatitis was proved (Balayan et al., 1983).

### Hepatitis G virus

Hepatitis G virus is also described, however it is still not known if it causes any diseases in humans (Hall, 2007).

Apart from viral hepatitis, several other causes of jaundice are also reported. These include malignancy, sepsis, shock, cirrhosis, gall stones, drugs, and autoimmune hepatitis (Whitehead et al., 2001).

## Ethnopharmacological approaches for management of jaundice: history, significance, and diversity of the used medicinal plants

According to the review by Hatfield (2004), several unusual practices have been employed to treat jaundice. In the British traditional medicine, there are some unusual home remedies for jaundice. A grotesque fallacy from Staffordshire is via the use of a bladder, which is filled by the patient's urine and placed close to the fire. It is believed that the patient recovers as it dries out. Another example of this kind is recorded from west Sussex, where an alive spider is rolled up in its own web and gulp down as a pill to cure jaundice. In Westmoreland alive head lice, and roasted powdered earthworms in Ireland, are ingested to cure jaundice (Hatfield, 2004). The use of snails was also found during seventeenth century as a cure for jaundice. Use of sheep's dung in water, giving the patient a violent fright (Hatfield, 2004) and a decoction of the common slater (woodlouse) in beer is also found to cure jaundice in Scotland. Urine was also used in various countries viz. in Ireland, urine mingled with milk like a drink against jaundice. In Yorkshire, baked rye cake with patient's urine under slow burning was believed to fade the illness away (Hatfield, 2004). In India such type of unusual practices were very common in the past. Certain people claimed to cure jaundice by spiritual practices and the use of *Mantras* (a sacred utterance, a spiritual sound, mainly are group of words in Sanskrit) along with some precautions, such as avoiding the fried and oily food, avoiding yellow color food especially use of turmeric in food. The practitioners used oil along with Durva [*Cynodon dactylon* (L.) Pers.], and used a bronze coin to treat the patients, for 3–4 weeks and within this time the patient gets rid from jaundice.

Several household remedies are used for jaundice treatment including eggs, cider, tea made from oats (*Avena* sp.) and vinegar. Some less distasteful propositions were, to use pearls dissolved in vinegar (De Lys, 1948) and a mixture of horn scraping and honey (Allen, 1963). Eating shellfish is a suggestion of Japanese origin (UCLA Folk Archives, record number 23_5423). Similarly to the Yorkshire method, baking of cornmeal cake with the patient's urine and its burning was used to cure jaundice (Hyatt, 1965; Hatfield, 2004). Various amulets are also used in North American folk medicine against jaundice. Some of these were made up of metals such as a copper necklace, or coin worn around the neck (UCLA Folklore Archives, record number 11_6404; Hatfield, 2004); gold piece of $ 5 strapped to the chest, or red beets worn round the neck (Puckett, 1981). Dressing of the patient in yellow clothes is a common practice in Pennsylvania to cure jaundice, which is in fact a custom of Russian origin (Crosby, 1927; Hatfield, 2004). Transference is also a typical example of jaundice cure, in which hard-boiled eggs strung over a necklace or placed under the armpits overnight (Hyatt, 1965; Hatfield, 2004). The next morning the egg white would be yellow, and the patient becomes white (Rogers, 1941). Some other sundry remedies for children suffering from jaundice are to place baby between two pillows, which would “bleach” out the jaundice (UCLA Folklore Archives, record number 23_58900, Hatfield, 2004). Although, these are based on typical practices used in older times, presumably these “treatments” do not work.

In Ayurveda, a variety of pathological conditions associated with jaundice are described under the generalized title referred as “*Pandu-roga*” (generally considered as anemia) which is a Sanskrit term meaning yellowish pale, or white disease. One of the important unique texts of Ayurveda, the *Charak Samhita*, or *Carak Samhita* (Before second Century A.D.) described in detail the eight categories of *Pandu-roga* (wherein another term “*Udarroga”* is mentioned for gastroenterological disorder) including “*Kamala*,” which seems to be the most relevant for jaundice condition. It was further described that *Kamala* (jaundice) is not developed during the initial stage of the condition. The occurrence of jaundice may develop if the initial *Pandu-roga* is not cured properly, and if the patient consumes bile-producing food. There are several important symptoms attributed to *Kamala*, which seem to be clearly associated with jaundice. They include: yellowish to greenish-yellow coloration of urine, sclera, nails and skin, reduction in the power of the senses, weakness, anorexia, indigestion, burning sensation and sometimes red urine (Durkin, 1988). *Charaka* (*Caraka*) described the treatment of various *Pandu-roga* including *Kamala* by different methods like emesis, evacuation of bowels, ingestion of medicated ghee, diet rich in rice, wheat, barley, and broth of *Mung* leaves (*Vigna radiata* L. R. Wilczek), as well as the use of different medications, which could restore humoral balance. All categories of *Pandu-roga* were mainly described as caused by the eating habits, and that the excess of hot, sour, salty, lentils, sesame oil, and seed pulp may cause various gastrointestinal problems. As the Ayurveda also tells about the life style, it was further depicted that overindulgence in sexual intercourse, suppression and negligence of bodily urges, emotional attributes (anger, sorrow, worry etc.) and day time sleeping may also cause an imbalance among the three humors (*Vata, Pitta, Kapha*, which are the basis of Ayurveda) by provoking bile in excess amount. Such types of imbalance showed different symptoms of jaundice and mainly the yellow pigmentation of skin. Some of the frequently used plants described include *Phyllanthus emblica* L., *Operculina turpethum* (L.) Silva Manso, *Azadirachta indica* A. Juss., *Zingiber officinale* Roscoe, *Piper longum* L., *P. nigrum* L., *Curcuma* spp. *Swertia chirayita* (Roxb.) Buch.-Ham. ex C.B.Clarke, *Picrorhiza kurroa* Royle ex Benth., *Terminalia bellirica* (Gaertn.) Roxb., and *T. chebula*. Most of these plants are already listed in Table 1, which indicates the significance of the Ayurvedic plants for the treatment of this condition. In Nepal it was described that some of the *Vaidya*s are being practicing Ayurved (Indian System of medicine). The *Vaidya*s of Kathmandu used to treat several ailments, although they are renowned for their ability to treat jaundice. Jaundice is considered as one of the most common disease conditions in Nepal and its association with viral hepatitis. An interesting fact reported by Durkin in 1988 is that, even though the people who generally do not rely on Ayurvedic treatment are also consulting to *Vaidya*, if jaundice occurs to them or their family members. It is also believed that effective treatment for jaundice is present in Ayurveda. The use of numerous plants in jaundice supports the above statement as Ayurveda utilizes a large number of medicinal plants and the major portion of Ayurvedic treatment relies on medicinal plant based formulations. The term *Kamala* and *Kamala pitta* are generally used by *Vaidya* in Nepal for jaundice. The cause of jaundice may include bile promoting food, dirty water intake from which the microbes could transmit and seasonal fluctuations (it was also observed that occurrence of jaundice is more frequent in autumn; Durkin, 1988).

**Table 1:** Overview of reported medicinal plants used to cure jaundice worldwide.

**Plant**	**Country**	**Formulation and references**	**Web hits (Google scholar)**
*Acacia concina* (Willd.) DC. (Mimosaceae)	India	An infusion mixture of leaves with black pepper and tamarind is taken orally (Sharma et al., 2001); leaves powder (Poonam and Singh, 2009)	84
*Acokanthera schimperi* (A. DC.) Schweinf (Apocynaceae)	Ethiopia	Ground leaves mixed with water (Teklehaymanot et al., 2007)	46
*Aconitum rotundifolium* Kar. & Kir. (Ranunculaceae)	India	Whole plant, root juice extracted by crushing is taken orally with equal volume of water (Singh and Lal, 2008)	12
*Acalypha fructicosa* Forssk.	India	Part used not indicated (Thambiraj and Paulsamy, 2011; Thambiraj et al., 2012; Seebaluck et al., 2015)	824
*Acalypha indica* L.	India	Leaves are used (Paindla and Mamidala, 2014; Seebaluck et al., 2015)	807
*Acalypha racemosa* Wall. ex Baill.	Nigeria	Leaves are used (Iniaghe et al., 2008, 2009; Seebaluck et al., 2015)	496
*Acalypha* torta Pax & K. Hoffm.	Nigeria	Part used not indicated (Onocha et al., 2011; Seebaluck et al., 2015)	31
*Adiantum capillus-veneris* L. (Pteridaceae)	Iran	Frond infusion (Amiri et al., 2014)	228
*Aerva lanata* (L.) Juss. ex Schult. (Amaranthaceae)	India	The root is crushed, squeezed, and the juice is used (Upadhyay et al., 2010)	376
*Agastache mexicana* (Kunth) Lint & Epling (Lamiaceae)	Mexico	Aerial part, flower infusion (Andrade-Cetto, 2009)	03
*Alhagi graecorum Boiss*.(Leguminosae/Fabaceae)	Iran	Aerial part Manna decoction soak (Amiri et al., 2014)	19
*Alhagi maurorum* Medik. (Leguminosae/Fabaceae)	Iran	Aerial part Manna decoction soak (Amiri et al., 2014)	111
*Aloe vera* (L.) Burm f. (Liliaceae)	India	The infusion of leaves is orally given twice a day for 10–12 days (Sharma et al., 2012)	2150
*Amaranthus spinosus* L. (Amaranthaceae)	India	The ash of fruits is administered orally two-three times a day for 2–3 weeks (Sharma et al., 2012)	605
*Amomum subulatum* Roxb. (Zingiberaceae)	India	Rhizome decoction (Sharma et al., 2001)	114
*Andrographis paniculata* (Burm. f.) Nees. (Acanthaceae)	India, Indo-Burma Hotspot	The paste of leaves is mixed with sugar, made into pills of 4 g each and given two times a day for 10–15 days (Sharma et al., 2012); Whole plant (Rai and Lalramnghinglova, 2011)	1450
*Anthocleista djalonensis* A Chev. (Loganiaceae)	Mali, (West Africa)	Leaves decoction (Togola et al., 2005)	61
*Ardisia japonica* (Thunb.) Blume (Myrsinaceae)	China	Whole plant (Hong et al., 2015)	60
*Ardisia paniculata* Roxb. (Primulaceae)	India (Indo-Burma Hotspot)	Crushed root in combination with *Smilax ovalifolia* Roxb. & *Bridelia tomentosa* Blume. is boiled in water (Rai and Lalramnghinglova, 2011)	13
*Argemone mexicana* L. (Papaveraceae)	India	Yellow sap and whole plant is used (Sharma et al., 2012; Mathur and Joshi, 2013)	731
*Artemisia abrotanum* L. (Asteraceae)	East and North Bosnia and Herzegovina	Aerial part is used (Šarić-Kundalić et al., 2011)	91
*Artemisia capillaris* Thunb. (Asteraceae)	China	Whole plant (Hong et al., 2015)	714
*Artemisia vulgaris* L. (Asteraceae)	East and north Bosnia and Herzegovina	Leaves are used (Šarić-Kundalić et al., 2011)	456
*Artemisia scoparia* Waldst. & Kitam. (Asteraceae)	China	Plant extract (Yeung et al., 1993)	370
*Asparagus racemosus* Willd. (Liliaceae)	India	The root is cut into pieces; gruel is prepared with rice and taken (Upadhyay et al., 2010)	1140
*Asphodelus microcarpus* Salzm. and Viv. (Liliaceae)	Israel	Bulb and root tincture is made for oral administration (Said et al., 2002)	30
*Astragalus fasciculifolius* subsp. *arbusculinus* (Bornm. & Gauba) Tietz (Leguminosae)	Iran	Gum decoction (Amiri et al., 2014)	02
*Averrhoa carambola* L. (Averrhoaceae)	India	The decoction of fruits is prescribed orally two–three times a day for 3 weeks (Sharma et al., 2012)	237
*Averrhoa* sp. (Oxalidaceae)	India (Indo-Burma Hotspot)	Three-four slices of fruits (Rai and Lalramnghinglova, 2011)	302
*Azadirachta indica* A. Juss (Meliaceae)	India	The decoction of bark is prescribed orally, two full teaspoons, twice a day for 2 weeks (Sharma et al., 2012)	2450
*Baliospermum solanifolium* (Burm.) Suresh (Euphorbiaceae)	India	Root powder is used (Sharma et al., 2012; Mathur and Joshi, 2013)	12
*Benincasa hispida* (Thunb.) Cogn. (Cucurbitaceae)	India	The juice of fruits (two-three teaspoons) is given orally twice a day for 2 weeks (Sharma et al., 2012)	240
*Berberis jaeschkeana* C.K. Schneid (Berberidaceae)	India	Root and fruits are used (Sharma and Samant, 2014)	10
*Berberis aristata* DC. (Berberidaceae)	Nepal	Leaf juice is taken orally (Rokaya et al., 2010)	639
*Berberis asiatica* Roxb. ex. DC. (Berberidaceae)	India	Fresh roots decoction is later filtered through a cloth, concentrated and dried in shade. Small pills (each of ca. 1–1.5 g) are made from this and are consumed with “*Kujja Mishri*” (local sweet made out of sugar) and water (Uniyal et al., 2006)	139
Berberis integrrima Bunge (Beberidaceae)	Iran	Fruit extracts (Amiri et al., 2014)	04
*Berberis vulgaris* L. (Berberidaceae)	Turkey	Fruit (Cakilcioglu et al., 2011)	573
*Betula utilis* D. Don (Betulaceae)	India	Bark, wood, leaf, root (Angmo et al., 2012; Sharma and Samant, 2014)	171
*Bidens andicola* H.B.K. (Compositae)	Peru	Decoction of leaves (Rehecho et al., 2011)	02
*Bidens pilosa* L. (Compositae)	China	Whole plant (Hong et al., 2015)	457
*Boerhavia diffusa* L. (Nyctaginaceae)	India	Whole plant (Mathur and Joshi, 2013); root extract (Sharma et al., 2012)	593
*Bromelia laciniosa* Mart. ex Schult. f. (Bromeliaceae)	Brazil	Flower, leaf, fruit (de Albuquerque et al., 2007)	06
*Cajanus cajan* (L.) Millsp. (Leguninosae)	India	The juice of leaves with honey is administered internally, twice a day for 15 days (Sharma et al., 2012)	585
*Canscora lucidissima* (Levl. et Vaniot) Hand.-Mazz (Gentianaceae)	China	Whole plant (Hong et al., 2015)	03
*Capparis spinosa* L.(Capparaceae)	Iran, India	Fruit decoction (Amiri et al., 2014); Dry powder of Green shoots is taken orally twice a day to cure liver pain (preliminary stage of jaundice; (Singh and Lal, 2008)	363
*Carica papaya* L. (Caricaceae)	India	Decoction of unripe fruit (Sharma et al., 2001)	1320
*Cassia fistula* L. (Leguminosae)	Iran, India	Fruit extract (Amiri et al., 2014); Fruit infusion (Sharma et al., 2012)	1200
*Cassytha filiformis* L. (Lauraceae)	China	Stem (Hong et al., 2015)	134
*Centella asiatica* (L.) Urb. (Apiaceae)	India	The leaf powder (3 g) along with goat milk is given orally once a day for about 8–10 days (Sharma et al., 2012)	1680
*Chonemorpha fragrans* (Moon) Alston (Apocynaceae)	Nepal	Leaves juice about 3 teaspoons twice a day (Malla et al., 2015)	18
*Cicer microphyllum* Benth. (Leguminosae)	India	Fruit, aerial part, leaf (Angmo et al., 2012; Sharma and Samant, 2014)	29
*Cichorium intybus* L. (Asteraceae)	Iran	Decoction of aerial part (Amiri et al., 2014)	725
*Cirsium japonicum* DC. var. *ussuriense* Kitamura (Asteraceae)	Korea, China	Fried dry vegetables leaf sprout, soup (Kim et al., 2006), root, whole plant (Hong et al., 2015)	36
*Cissampelos pareira* L. (Menispermaceae)	India	The half teaspoon juice of fresh leaves is used internally twice a day for 12–15 days (Sharma et al., 2012)	451
*Cistanche tubulosa* (Schenk.) Hook. f. (Orobanchaceae)	Israel	Decoction of 30 g leaves and flowers in water is taken orally, one cup/day until the condition improves (Said et al., 2002)	45
*Clematis chinensis* Osbeck. (Ranunculaceae)	China	Root, leaf (Hong et al., 2015)	193
*Cochlospermum tinctorium* A. Rich. (Cochlospermaceae)	Mali, (West Africa)	Leaves and roots (Togola et al., 2005)	75
*Coriandrum sativum* L.(Apiaceae)	Iran	Aerial decoction (Amiri et al., 2014)	739
*Costus speciosus* (Koenig.) Sm. (Zingiberaceae)	India	The roots are soaked in water for few hours, boiled and decoction half teaspoon is recommended, once a day for 2 weeks (Sharma et al., 2012)	507
*Cotoneaster nummularius* Fisch. & C.A.Mey. (Rosaceae)	Iran	Manna soak (Amiri et al., 2014)	06
*Crepis flexuosa* (DC.) Benth. (Asteraceae)	India	Fresh juice mixed in equal proportion with water is taken once a day (Singh and Lal, 2008)	07
*Cucumis dispsaceus* Ehernb. Ex. Spach, (Curcurbitaceae)	Ethiopia	Ground root mixed with water (Teklehaymanot et al., 2007)	02
*Curculigo orchiodis* Haertn. (Amaryllidaceae)	India	Root tubers (Parveen et al., 2007)	02
*Curcuma zedoaria* (Christm.) Rosc. (Zingiberaceae)	India	Rhizome crushed in water (Poonam and Singh, 2009)	259
*Curcuma aromatica* Salisb. (Zingiberaceae)	China	Tuber (Hong et al., 2015)	369
*Cuscuta chinensis* Lam. (Convalvulaceae)	Thialand	Leaf/stem decoction (Panyaphu et al., 2011)	86
*Cuscuta reflexa* Roxb. (Convolvulaceae)	Nepal, India	Whole plant is cut into pieces, or crushed, decocted, and the liquid is taken orally (Rokaya et al., 2010; Malla et al., 2015); whole plant decoction and seed paste (Sharma et al., 2012)	627
*Cynara scolymus* L.*(Asteraceae)*	Iran	Aerial decoction (Amiri et al., 2014)	293
*Cynodon dactylon* (L.) Pers. (Poaceae)	India	The juice of leaves one-two teaspoons are taken, twice a day for a week (Sharma et al., 2012)	1070
*Cyperus rotundus* L. *(Cyperaceae)*	Iran	Root decoction (Amiri et al., 2014)	797
*Datura stramonium* L. (Solanaceae)	India	Leaf, seed, fruit (Sharma and Samant, 2014)	854
*Daucus carota* L. (Apiaceae)	Israel	Root juice is taken and taken 2–3 times a day (Said et al., 2002)	628
*Dendrocnide sinuata* (Blume) Chew (Urticaceae)	India (Indo-Burma Hotspot)	The roots are boiled along with crabs and the water is taken (Rai and Lalramnghinglova, 2011)	15
*Descurainia sophia* (L.) Webb ex Prantl (Brassicaceae)	Iran	Seeds soaked (Amiri et al., 2014)	71
*Desmostachya bipinnata* (L.) Stapf (Poaceae)	Nepal	Root juice about four teaspoons three times a day (Malla et al., 2015)	150
*Dichondra repens* J.R. Forst. & G. Forst (Convolvulaceae)	China	Whole plant (Hong et al., 2015)	26
*Dillenia indica* L. (Dilleniaceae)	India (Indo-Burma Hotspot)	The fruit is boiled and the water is taken (Rai and Lalramnghinglova, 2011)	238
*Descurainia sophia* (L.) Webb ex Prantl (Brassicaceae)	Iran	Seed soak is used (Amiri et al., 2014)	73
*Ecballium elaterium* A.Rich. (Cucurbitaceae)	Jordan, Israel	Nasal drops of fruit juice for infantile jaundice (Aburjai et al., 2007); fruit juice (Said et al., 2002)	142
*Eclipta prostrata* (L.) L. [formerly *Eclipta alba* (L.) Hassk.] (Esteraceae)	India	The leaves are crushed finely and soaked for a night; this water is taken once per day in the morning or twice a day for 3–4 weeks (Sharma et al., 2012); leaf, whole plant juice (Parveen et al., 2007; Mathur and Joshi, 2013)	560
*Ehretia laevis* Roxb. (Ehretiaceae)	India	Soaked seeds are made into paste, mixed with powder of badi elaichi (*Amomum subulatum*) and given orally with milk, three times a day (Sharma et al., 2012)	40
*Elephantopus scaber* L. (Asteraceae)	China	Whole plant (Hong et al., 2015)	305
*Embelia ribes* Burm.f. (Primulaceae)	Iran	Fruit decoction (Amiri et al., 2014)	333
*Eupatorium chinense* L. var. *simplicifolium* (Asteraceae)	Korea	Seasoned cooked vegetables sprouts (Kim et al., 2006)	07
*Fibraurea recisa* Pierre (Menispermaceae)	China	Root (Hong et al., 2015)	09
*Ficus religiosa* L. (Moraceae)	India	The decoction of stem bark is recommended orally twice a day for a week (Sharma et al., 2012), two-three leaves of *Ficus* with two leaves of *Azadiracta*, kept in a betel leaf and given for chewing to jaundice patient as remedy (Upadhyay et al., 2010)	715
*Ficus tikoua* Bureau	China	Whole plant (Hong et al., 2015)	03
*Flacourtia indica* (Burm. F.) Merr. (Flacourtiaceae)	India	Root paste (Upadhyay et al., 2010); Decoction of leaves and fruits (Sharma et al., 2012)	192
*Fumaria vaillantii* Loisel. (Papaveraceae)	Iran	Aerial part infusion (Amiri et al., 2014)	29
*Galium rotundifolium* L. (Rubiaceae)	India	Whole plant (Sharma and Samant, 2014)	06
*Gardenia jasminoides* J. Ellis	China	Fruit (Hong et al., 2015)	452
*Gentiana moorcroftiana* Wall. Ex G. Don (Gentianaceae)	India	Juice extracted by crushed fresh aerial parts is taken on an empty stomach to cure jaundice (Singh and Lal, 2008)	06
*Gentiana tubiflora* (G.Don) Griseb. (Gentianaceae)	India	The fresh juice of aerial parts is mixed with equal quantity of water and about half glass of the mixture is taken orally during the morning hours (Singh and Lal, 2008)	10
*Gentianopsis detonsa* (Rottb.) Ma. (Gentianaceae)	India	Extracted fresh juice is taken orally in jaundice (Singh and Lal, 2008)	07
*Geranium pratense* Linn. (Geraniaceae)	India	Whole plant, one spoon of powder is taken orally with water (Singh and Lal, 2008)	33
*Glechoma hederacea* L. (Lamiaceae)	East and North Bosnia and Herzegovina	Aerial part, leaf is used; (Šarić-Kundalić et al., 2011)	89
*Glechoma hirsuta* Waldst. Et Kit. (Lamiaceae)	East and north Bosnia and Herzegovina	Aerial part, leaf (Šarić-Kundalić et al., 2011)	01
*Glycosmis pentaphylla* (Retz.) DC. (Rutaceae)	India	The juice of leaves is given, half teaspoon three times a day for 15 days (Sharma et al., 2012)	218
*Glycyrrhiza* spp.	China	Prescribed in combination, the active phytochemical is utilized (Fok, 2001)	2860
*Gossypium barbadense* L. (Malvaceae)	Brazil	Leaf (de Albuquerque et al., 2007)	92
*Gynura conyza* Cass. (Asteraceae)	India	Leaf decoction (Sharma et al., 2001)	04
*Haldina cordifolia* (Roxb.) Ridsdale (Rubiaceae)	India	The decoction of bark is taken orally (Sharma et al., 2012)	53
*Hibiscus rosa-sinensis* L. (Malvaceae)	India (Indo-Burma Hotspot)	The raw flower is taken (Rai and Lalramnghinglova, 2011)	541
*Hippocratea africana* (willd.) Loes ex Engl. (Celastraceae)	Nepal, Nigeria	Root boiled in palm wine, macerated in local gin or soda water (Ajibesin et al., 2008; Rokaya et al., 2010)	09
*Hippophae rhamnoides* Linn. (Elaeagnaceae)	India	Juice extracted from fruit pulp is boiled (low temperature) with half liter of water till it gets solidified. Tablets are made and 2 tablets are given to women with milk to cure excessive bleeding and jaundice (Singh and Lal, 2008)	238
*Hippophae tibetana* Schlecht. (Elaeagnaceae)	India	Dried fruits are crushed and boiled in water to prepare a decoction. The decoction obtained is taken to cure jaundice (Singh and Lal, 2008)	34
*Holarrhena pubescens* Wall. (Apocynaceae)	India	The seeds are crushed, soaked into water for a night; dried in shade made into powder, about 2–3 g of which is taken with lukewarm water twice for 16 days (Sharma et al., 2012)	216
*Inula cappa* (Buch.-Ham. ex D. Don) DC. (Asteraceae)	India (Indo-Burma Hotspot)	The leaves are crushed with those of *Plantago erosa* Wall. ex Roxb. & *Lobelia angulata* G. Forst. and the juice is taken orally three times daily for jaundice (Rai and Lalramnghinglova, 2011)	47
*Ipomoea purpurea* (L.) Roth. (Convolvulaceae)	Nepal	A decoction of the leaves about two teaspoons twice a day (Malla et al., 2015)	45
*Juncus effusus* L.(Juncaceae)	China	Whole plant (Hong et al., 2015)	34
*Lagenaria siceraria* (Molina) Standley (Cucurbitaceae)	India	Leaf, seed, root (Parveen et al., 2007)	336
*Lagerstroemia speciosa* (L.) Pers. (Lythraceae)	India (Indo-Burma Hotspot)	Root decoction (Sharma et al., 2001; Rai and Lalramnghinglova, 2011)	180
*Lannea acida* A.Rich. (Syn. *Lannea microcarpa* Eng & Kr. Are) (Anacardiaceae)	Burkina Faso	Decoction of leaf and stem bark (Nadembega et al., 2011)	20
*Laportea crenulata* Gaud. (Urticaceae)	India	Root decoction (Sharma et al., 2001)	25
*Lawsonia inermis* L. (Lythraceae)	India	Root decoction (Sharma et al., 2012); One gram of fresh leaves and 3 g black peppers are made into paste in 50 ml of cow's milk and it is taken for 1 month (Parveen et al., 2007; Upadhyay et al., 2010)	869
*Leptadenia pyrotechnica* (Forsk.) Decne (Apocynaceae)	India	Whole plant (Upadhyay et al., 2010)	77
*Leucas aspera* Spreng (Lamiaceae)	India	Juice of leaves and flowers mixed with milk is used (Parveen et al., 2007)	561
*Lippia gracilis* Schauer (Verbenaceae)	Brazil	Leaf (de Albuquerque et al., 2007)	17
*Lobelia angulata* G. Forst. (Campanulaceae)	India (Indo-Burma Hotspot)	The whole plant is crushed with *Plantago erosa* Wall. ex Roxb. and *Inula cappa* (Buch.-Ham. ex D. Don) DC. & the juice is taken orally three times daily for diabetes & jaundice (Rai and Lalramnghinglova, 2011)	08
*Lonicera japonica* Thunb. (Caprifoliaceae)	China	Stem (Hong et al., 2015)	253
*Luffa acutangula* (L.) Roxb. (Cucurbitaceae)	India	Leaves, stem, and seeds are crushed, strained in cloth and inhaled by jaundice patient (Upadhyay et al., 2010)	233
*Lysimachia christinae* Hance (Primulaceae)	China	Whole plant (Hong et al., 2015)	41
*Marsilea quadrifolia* L. (Marsileaceae)	China	Whole plant (Hong et al., 2015)	89
*Mallotus roxburghianus* Müll. Arg. (Euphorbiaceae)	India (Indo-Burma Hotspot)	Bark is used to treat jaundice (Rai and Lalramnghinglova, 2011)	08
*Malva sylvestris* L. (Malvaceae)	Iran	Flower infusion (Amiri et al., 2014)	206
*Malva verticillata* L. (Malvaceae)	Korea	Leaf soup, clear soup with flour dumpling in it (Kim et al., 2006)	78
*Mangifera indica* L. (Anacardiaceae)	India, Nepal	The decoction of bark/ cotyledon is administered orally, thrice a day for 15–20 days (Joshi and Joshi, 2000; Sharma et al., 2012)	1,320
*Momordica charantia* L. (Cucurbitaceae)	India	The juice of fruits is administered internally twice a day for 2 weeks (Sharma et al., 2012); leaf juice with the fruits of *Terminalia chebula* Retz. (Sharma et al., 2001)	1,370
*Nandina domestica* Thunb. (Berberidaceae)	China	Root and stem decoction (Hong et al., 2015)	38
*Musa superba* Roxb. (Musaceae)	India	Stem juice (Sharma et al., 2001)	13
*Nelumbo nucifera* Gaertn. (Nymphaeaceae)	India	Stem bark extract (Upadhyay et al., 2010)	391
*Nephrolepis cordifolia* (L.) C. Presl (Davalliaceae)	China	Decoction of rhizome, Leaf, Whole plant (Hong et al., 2015)	77
*Nerium oleander* L. (Apocynaceae)	Israel	A standard decoction from wooden stem is taken orally (Said et al., 2002)	444
*Ocimum americanum* L. (Lamiaceae)	India	The decoction of whole plant is taken internally, thrice a day for 3–4 weeks (Sharma et al., 2012)	160
*Oroxylum indicum* (L.) Kurz (Bignoniaceae),	India, China	The crushed bark is soaked into water in an earthen pot for a night, given orally in early morning or one to two times a day for 1 week (Sharma et al., 2012), seed (Hong et al., 2015)	505
*Oxalis corniculata* L. (Oxalidaceae)	India	The juice of leaves is given orally, twice a day for 1–2 weeks (Sharma et al., 2012)	715
*Passiflora* spp. (Passifloraceae)	India	Inner part of fruits (Sharma et al., 2001)	627
*Pavetta indica* L. (Rubiaceae)	India (Indo-Burma Hotspot)	Roots are used (Rai and Lalramnghinglova, 2011)	99
*Peganum harmala* L. (Zygophyllaceae)	India	Seed powder (Parveen et al., 2007)	378
*Peumus boldus* Molina (Monimiaceae)	Chile	Leaves are used (Duke, 2002)	99
*Phyllanthus amarus* Schumach. & Thonn.(Euphorbiaceae)	India	The infusion of whole plant is taken twice a day for 4 weeks (Sharma et al., 2012)	1190
*Phyllanthus emblica* L. (Phyllanthaceae)	Iran, India	Fruit decoction (Sharma et al., 2012; Mathur and Joshi, 2013; Amiri et al., 2014)	1090
*Phyllanthus fraternus* G.L. Webster. (Phyllanthaceae)	India	Fresh root (Mathur and Joshi, 2013)	271
*Phyllanthus niruri* Linn. (Euphorbiaceae)	India	Fresh roots juice (Poonam and Singh, 2009)	958
*Phyllanthus urinaria* L.	China	Whole plant (Hong et al., 2015)	416
Physalis alkekengi L.(Solanaceae)	Iran	Fruit decoction (Amiri et al., 2014)	79
*Physalis divaricata* D. Don (Solanaceae),	India, China	The root juice, two teaspoons two times a day for 4 weeks (Sharma et al., 2012), whole plant (Hong et al., 2015)	14
*Picrorhiza kurrooa* Royle ex Benth. (Scrophulariaceae)	India	Root, leaf, rhizome, stem (Uniyal et al., 2006; Sharma and Samant, 2014)	879
*Pilosocereus gounellei* (F.A.C.Weber ex K.Schum.) Byles & G.D.Rowley (Cactaceae)	Brazil	Cladode, flower, root (de Albuquerque et al., 2007)	04
*Piper betle* L. (Piperaceae)	India	Two leaves of *Azadiracta* and two to three leaves of Peepal (*Ficus religosa*) are kept in a betel leaf and given for chewing to jaundice patient (Upadhyay et al., 2010)	363
*Pistacia lentiscus* L. (Anacardiaceae)	Israel	An infusion of 50 g leaves is soaked in water for 24 h and taken orally (Said et al., 2002)	253
*Plantago asiatica* subsp. *erosa* (Wall.) Z. Yu Li (Previously *Plantago erosa* Wall. ex Roxb.) (Plantaginaceae)	India (Indo-Burma Hotspot)	The leaf & whole plant are crushed with *Lobelia angulata* G. Forst. and *Inula cappa* (Buch.-Ham. ex D. Don) DC. and the juice is taken orally three times daily (Rai and Lalramnghinglova, 2011)	97
*Plantago major* L. (Plantaginaceae)	Iran	Seeds soaked (Amiri et al., 2014)	506
*Plantago ovata* Forssk. (Plantaginaceae)	Iran	Seeds soaked (Amiri et al., 2014)	490
*Polygonum perfoliatum* (L.) L (Polygonaceae)	China	Whole plant (Hong et al., 2015)	113
*Polygonum tortuosum* D. Don. (Polygonaceae)	India	Powder obtained from aerial parts is consumed orally with water (Singh and Lal, 2008)	11
*Polypodium vulgare* L.(Polypodiaceae)	Iran	Rhizome infusion (Amiri et al., 2014)	76
*Portulaca oleracea* L. (Portulacaceae)	Iran	Seeds soaked (Amiri et al., 2014)	513
*Pteris multifida* Poir.(Pteridaceae)	China	Whole plant (Hong et al., 2015)	73
*Reynoutria japonica* Houtt (Polygonaceae)	China	Rhizome (Hong et al., 2015)	46
*Rheum officinale* Baill. (Polygonaceae)	Jordan	Root (Lev and Amar, 2002)	259
*Rheum palmatum* L. (Polygonaceae)	Iran	Root decoction (Amiri et al., 2014)	500
*Rheum ribes* L. (Polygonaceae)	Iran	Fruit infusion (Amiri et al., 2014)	44
*Rheum turkestanicum* Janisch. (Polygonaceae)	Iran	Root extract (Amiri et al., 2014)	02 (01-same reference)
*Rhus coriaria* L. (Anacardiaceae)	Iran	Fruit infusion (Amiri et al., 2014)	88
*Rosa sericea* Lindl. (Rosaceae)	Nepal	Flower and fruit powder is used (Rokaya et al., 2010)	28
*Rosa webbiana* Wall. ex Royle (Rosaceae)	India	Fruit, flower, aerial part (Angmo et al., 2012; Sharma and Samant, 2014), juice extracted from the flowers, is mixed with small quantity of water (Singh and Lal, 2008)	56
*Rubus parvifolius* L.(Rosaceae)	China	Whole plant (Hong et al., 2015)	32
*Rumex acetosella* L. (Polygonaceae)	Iran	Aerial part infusion (Amiri et al., 2014)	144
*Saccharum officinarum* L. (Poaceae)	India	Plant juice and jaggery are given orally to jaundice patient (Upadhyay et al., 2010)	380
*Salix alba* L.(Salicaceae)	Iran	Leaf decoction (Amiri et al., 2014)	242
*Salix excelsa* J.F.Gmel. *(Salicaceae)*	Iran	Manna soak (Amiri et al., 2014)	02
*Salvia macrosiphon* Boiss. *(Lamiaceae)*	Iran	Seed infusion (Amiri et al., 2014)	08
*Scoparia dulcis* L. (Plantaginaceae)	India (Indo-Burma Hotspot)	Crushed whole plant juice (Rai and Lalramnghinglova, 2011)	424
*Senecio scandens* Buch.-Ham. ex D. Don (Asteraceae)	China	Whole plant (Hong et al., 2015)	107
*Scorzonera divaricata* Turcz. (Asteraceae)	India	Leaf decoction prepared at low temperature is consumed orally to cure dysentery and jaundice (Singh and Lal, 2008)	07
*Sigesbeckia orientalis* L. (Asteraceae)	China	Whole plant (Hong et al., 2015)	75
*Silybum marianum* (L.) Gaertn. (Asteraceae)	Iran	Seed decoction (Amiri et al., 2014)	1160
*Solanum americanum* Mill. (Solanaceae)	India	The decoction of whole plant is used internally, thrice a day for 3 weeks (Sharma et al., 2012)	91
*Solanum incanum* L. (Solanaceae)	India	The fruits are crushed and soaked in water for a whole night and the water is taken in early morning (Sharma et al., 2012)	150
*Solidago decurrens* Lour. (Asteraceae)	China	Whole plant decoction taken orally (Hong et al., 2015)	08
*Solidago virga-aurea* Auct. var. *asiatica* (Asteraceae)	Korea	Seasoned cooked vegetables sprouts (Kim et al., 2006)	21
*Sonchus oleraceus* L. (Asteraceae)	India	Flower, aerial part, leaf (Sharma and Samant, 2014)	153
*Sphaeranthus indicus* L. (Asteraceae)	India	The expressed juice of plant (Upadhyay et al., 2010)	343
*Sphaeranthus senegalensis* DC. (Asteraceae)	India	Whole plant powder with sugar; 2–3 g of this preparation is taken with lukewarm water, twice a day for 2 weeks (Sharma et al., 2012)	24
*Spinacia oleracea* L. (Amaranthaceae)	Jordan	Seed (Lev and Amar, 2002)	165
*Striga asiatica* (L.) Kuntze (Scrophulariaceae)	China	Whole plant decoction taken orally (Hong et al., 2015)	154
*Tamarindus indica* L. (Leguminosae)	Iran	Fruit decoction (Amiri et al., 2014)	1030
*Tanacetum vulgare* L. (Compositae)	Ireland	Infusion (Allen and Hatfield, 2004)	183
*Taraxacum officinale* Wigg. (Asteraceae)	India, Nepal, China	Root (Joshi and Joshi, 2000); leaf, flower (Singh and Lal, 2008); tea prepared from *T. officinale* (Saroya, 2011)	721
*Terminalia chebula* Retz. (Combretaceae)	Iran	Fruit decoction (Amiri et al., 2014)	1460
*Teucrium chamardrys* L. (Lamiaceae)	Israel	Foliage (Said et al., 2002)	01
*Tinospora sinensis* Lour. Merr. [*Tinospora cordifolia* (Willd.) Miers Ex Hook F. and Thomas] (Menispermaceae)	India, Indo-Burma Hotspot	Stem juice is valued in high fever and given in jaundice either alone, or mixed with honey (Parveen et al., 2007; Upadhyay et al., 2010; Sharma et al., 2012); fruits (Rai and Lalramnghinglova, 2011); fruits paste (Poonam and Singh, 2009)	153 as *T. sinensis* and 1520 as (*T. cordifolia)*
*Toddalia asiatica* (L.) Lam. (Rutaceae)	Iran	Fruit decoction (Amiri et al., 2014)	193
*Tribulus terrestris* L.(Zygophyllaceae)	Iran, India	Aerial decoction (Amiri et al., 2014); Leaf juice (Sharma et al., 2012)	928
*Trichilia emetica* Vahl. (Meliaceae)	Mali, (West Africa)	Root bark (Togola et al., 2005)	63
*Trichosanthes cucumerina* L. (Cucurbitaceae)	India	Fruits infusion (Sharma et al., 2012)	123
*Trigonella emodi* Benth. (Leguminosae)	India	One spoon of powder is taken twice a day for a week to cure jaundice (Singh and Lal, 2008)	12
*Urtica dioica* L. (Urticaceae)	India	Leaf (Sharma and Samant, 2014)	706
*Uncaria rhynchophylla* (Miq.) Miq. ex Havil.(Rubiaceae)	China	Hooked stem grinded decoction (Hong et al., 2015)	57
*Uvaria chamae* P. Beauv. (Annonaceae)	Brazil, Nigeria	Root crushed and boiled in water or palm wine as decoction or macerated in soda water as infusion (de Albuquerque et al., 2007; Ajibesin et al., 2008)	57
*Veronica chamaedrys* L. (Plantaginaceae)	Ireland	Boiled leaves and stems (Allen and Hatfield, 2004)	10
*Viola inconspicua* Blume (Violaceae)	China	Whole plant decoction taken orally (Hong et al., 2015)	06
*Viola odorata* L. (Violaceae)	Iran	Flower infusion (Amiri et al., 2014)	263
*Vitex negundo* L. (Verbenaceae)	India	Leaf infusion (Sharma et al., 2012)	1090
*Woodfordia fruticosa* (L.) Kurz (Lythraceae)	India	The decoction of fruits is recommended orally (Sharma et al., 2012)	383
*Ziziphus jujuba* Miller (Rhamnaceae)	Iran	Fruit decoction (Amiri et al., 2014), Fruit extract (Ebrahimimd et al., 2011) (clinical study)	196

Various herbal medicines for treatment of jaundice are being used from ancient times. Combination of Bogbean (*Menyanthes trifoliate* L.), raspberry (*Rubus* sp.) and wild mint (*Mentha* spp.) was used to treat jaundice (Beith, 1995; Hatfield, 2004). In Shetland, Bogbean is known as gulsa girse, which literally means “yellow sickness plant” (Vickery, 1995). The bark decoction of another important plant, Barberry (*Berberis vulgaris* L.) also known as the “jaundice tree” in Cornwall; has been widely used for the treatment of jaundice in England and Ireland (Vickery, 1995). Some other important plants used for jaundice treatment are: dandelion (*Taraxacum campylodes* G.E. Haglund), *Ulex europaeus* L. (Vickery, 1995; Hatfield, 2004), and nettle roots (probably *Urtica dioca*; Hatfield, 2004). Greater celandine (*Chelidonium majus* L.) is used for the treatment of infantile jaundice. Other plants used mainly in European countries for the treatment of jaundice are Primarose (*Primula vulgaris* Huds.) in Ireland, cowslip (*Primula veris* L.), in England, leaves of the savage tree (*Juniperus* sp.) in Wales, (EFS record number 221), chickweed (*Tragopogon porrifolius* L.), salsify [*Stellaria media* (L.) Vill.] in East England, *Ulmus* spp. boiled in milk in Herefordshire and Ireland (Vickery, 1995; Hatfield, 2004).

In Britain other herbal remedies are used like dandelion (*Taraxacum* sp.), greater celandine, which is either worn in the shoes (Lick and Brendle, 1923) or ingested (Meyer, 1985). Various botanical remedies like walnut bark (*Juglans* sp.), boneset (*Eupatorium perfoliatum* L.), hops (*Humulus lupulus* L.), wild cherry bark (*Prunus serotina* Ehrh.), black alder [*Ilex verticillata* (L.) A. Gray], leaves or bark of peach [*Prunus persica* (L.) Batsch], cinquefoil (*Potentilla Canadensis* L.) (Meyer, 1985), strawberry leaves (*Fragaria* spp.) (Lick and Brendle, 1923), a tea made from catnip (*Nepeta cataria* L.) (Puckett, 1981) or from mullein (*Verbascum* sp.) (Parler, 1962) are used to treat jaundice. Some other important herbal infusion which are used to treat jaundice are yarrow (*Achillea millefolium* L.) (Hendricks, 1959), crossvine (*Bignonia capreolata* L.) (Rogers, 1941), calamus root (*Acorus calamus* L.) (Puckett, 1981), mayapple root (*Podophyllum peltatum* L.) (Clark, 1970), yellow dock root (*Rumex crispus* L.) (Lick and Brendle, 1923), rosemary leaves (*Rosmarinus officinalis* L.) (UCLA Folklore Archives, record number 4_2092), ironwood bark (*Carpinus caroliniana* Walter), and St. John's wort (*Hypericum perforatum* L.) (Hatfield, 2004). In Carolina, tea brewed from wild oranges and basil (*Ocimum basilicum* L.) (Brown, 1952-1964) was used for treating jaundice. In the Midwest, bruised lobelia and red pepper pods in whisky were used (Pickard and Buley, 1945). In Indiana a mixture containing bitter root (*Lewisia* sp.), red sumac (*Rhus* sp.), wild cherry bark (*Prunus* sp.), sarsaparilla root, and wild poplar root (*Populus* sp.) was used (Halpert, 1950; Hatfield, 2004). *Haematoxylon* spp. (longwood) has been used to treat jaundice in Mexico. The *Haematoxylon* spp. extract is utilized by two ways drinking the liquid extract, or by placing the glassful of extract on the window still for patient to gawk; further it was hoped to transfer the yellow color from the patient to the liquid (Curtin, 1907; Hatfield, 2004). Vegetables viz. collards (*Brassica oleracea* L.) (Browne, 1958), artichokes (*Helianthus tuberosus* L.) were also found useful in jaundice treatment, (UCLA Folklore Achieves, record number 6_7607). The use of tobacco (*Nicotiana* spp.) alleviate jaundice (Smith, 1929) and daisies (*Bellis perennis* L.) is recommended for the re-coloration after jaundice treatment (Clark, 1970). Native American practices are also rich in terms of plants for hepatic disorder. For example the use of *Juglans cinerea* L. is recommended from Iroquois (Herrick, 1977; Hatfield, 2004).

There is an emerging interest in the use of medicinal plants that have been used traditionally for treating various diseases. This is because medicinal plants have the lesser adverse effects and fewer complications compared to synthetic drugs. This is an important advantage of plant derived drugs, which is also advised by the World Health Organization (WHO). The search of bilirubin reducing substances of herbal origin has gained particular interest in recent times (Dennery, 2002; Wong et al., 2005). A number of plant derived products are used either alone, or in combination with various other modern treatment such as exchange transfusion or the phototherapy of infants with high bilirubin concentration (Ebrahimimd et al., 2011). Systematic scientific studies are required to evaluate the safety, efficacy and toxicity profile of various herbal drugs (Kunwar et al., 2009). The utilization of the medicinal plants in the treatment of jaundice is also evident from the number of case series, which exhibited faster reduction of jaundice than western medicine treatment (Ebrahimimd et al., 2011).

The induction of glutathione S-transferase and UDP-glucouronyl transferase (UGT) and the effect on bilirubin metabolism in rats was reported upon treatment with *Scutellaria, Rheum officinale* L., *Artemesia*, and *Gardenia*. The combination of extracts from the above mentioned plants is known as “Yin Zhi Huang” in the Chinese traditional medicine and is used in Asia for the management and treatment of neonatal jaundice via enhancement of bilirubin clearance (Yang and Lu, 1984; Chen and Guan, 1985; Dong et al., 1992; Elferink, 2004). Various medicinal plants have also been used from an ancient time in different countries, including Iran, China, Ethiopia, Mexico, West Africa, Turkey, Peru, Nepal etc., but there are still more studies required to prove their efficacy. Clinical studies have also been carried out in Iran to evaluate the effect of aqueous extract of *Zizyphus jujuba* Mill. on the neonatal jaundice. The results showed a straight effect of the plant extract on the neonates. The use of medicinal plants in the treatment of jaundice showed in some studies more potent stimulatory effect on bilirubin clearance when compared to phenobarbital (Yin et al., 1993; Zhao et al., 2006). Some of the plants such as *Z. jujuba* are used in the reinforcement of liver function during jaundice (Chan, 1994; Huang, 1998; Ebrahimimd et al., 2011). However there are some limitations present in the study carried out with *Z. jujuba* as it was shown effective in the treatment against neonatal jaundice during the first 12 h, but it was not found effective in the consecutive days (Ebrahimimd et al., 2011). The use of herbal medicines to heal the jaundice was also reported from Mashhad city in Iran, where the disease was found to be major health problem (Amiri et al., 2014).

**Figure 3:**
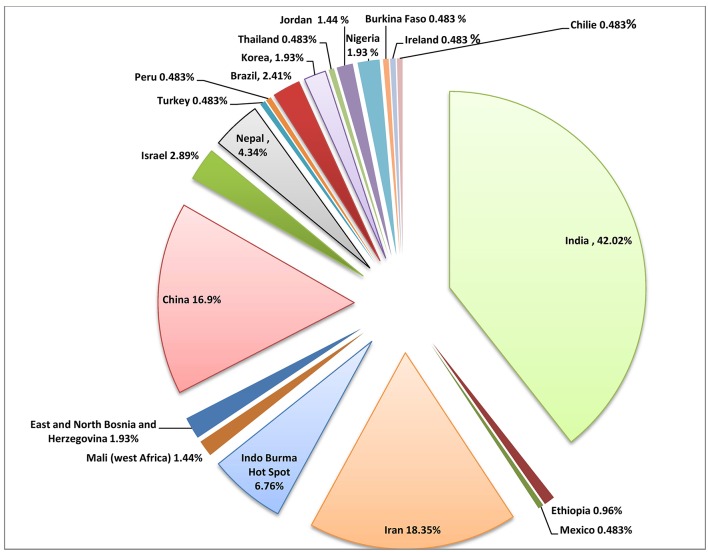
Pie chart showing the distribution of the ethnopharmacologically important plants for the treatment of jaundice worldwide.

There are two widely accepted methods of neonatal jaundice treatment, phototherapy and blood exchange (Amiri et al., 2014; Chen et al., 2017). In addition, herbal remedies are also very popular in the treatment of jaundice, especially in the Iranian tradition (Rajaei and Mohamadi, 2012; Amiri et al., 2014). A number of plant species have been utilized in Iran (Table 1 and Figure 3). There are very limited pharmacological interventions, which are used for the treatment of neonatal jaundice. The available pharmacological approaches for treatment of neonatal jaundice are based on different mechanisms of action. Metalloporphyrins, for example act via inhibition of heme oxygenase—the rate limiting enzyme in bilirubin production. The metalloporphyrin consists of a biocompatible metal ion, which does not degrade in tissues and is not photochemically active. Some of the important tissue heme oxygenase inhibitors are chromium mesoporphyrin (CrMP) and tin mesoporphyrin (SnMP) (Vreman et al., 1993; Dennery, 2002). Although, the metalloporphyrin are used frequently and are highly effective, some of those compounds are photochemically active (Dennery, 2002; Lee et al., 2017). Some of the studies revealed a dosage dependent mortality associated with SnMP application in neonatal Wistar rats (Hintz et al., 1990), moreover lipid peroxidation and heamolysis of RBCs was also reported (Keino et al., 1990). Some other adverse effect such as arrhythmia, tachyphylaxis (which is due to the induction of HO-1 protein and mRNA) etc. were also reported upon metalloporphyrins use. Though CrMP does not possess phototoxicity, it cannot be considered as safe because of the carcinogenic effect of Cr metal (De Flora et al., 1980; Voitkun et al., 1998; Dillon et al., 2000). Another important therapeutic regimen for neonatal jaundice is D-Penicillamine. The drug is used to cure neonatal jaundice mainly in Europe but not in the USA (Dennery, 2002). Its main mechanism of action is by the inhibition of HO activity (Juckett et al., 1998). Still there are risks of fatalities associated with this drug, along with the onset of aplastic anemia, thrombocytopenia, myasthenia gravis and Goodpasture syndrome (Louie et al., 1986; Peces et al., 1987; Fishel et al., 1989; Kaufman et al., 1996; Narayanan and Behari, 1999; Teive et al., 2017).

An extensive review has been carried out to explore the use of medicinal plants for jaundice treatment. A total of 207 plant species have been documented and presented in Table 1. The botanical name of the plant, family with the country name from where the plant is reported followed by formulation used for jaundice are summarized and web hits from Google scholar were gathered through Boolean information retrieval method using plant name with “AND” operator (Pohl et al., 2010).

Our work is the first review, which presents comprehensive documentation of the ethnomedicinal uses of the plants for the prevention and treatment of jaundice. Large number of medicinal plants has been shown to have potential in the development of approaches to obtain therapeutic regimen for the treatment of jaundice, although some of the molecules like sylimarin and andrographolide are already well established and widely used for the management of hepatic disorders worldwide. Many medicinal plants are being used from a long back in several countries. Graphical representation at Figure 3 shows the number of medicinal plants used for jaundice in 20 different countries/region, which have been documented in the review.

## Conclusion

Various new chemical entities have been developed from natural products, which have been filed in last few decades. Ethnopharmacological information is of prime importance for discovery and usage of significant therapeutically important molecules either obtained from natural sources, or inspired by nature. In this first part of our review, we discussed the history, metabolism process; some unusual practices used in older times and a comprehensive detail about 207 plants, which are used in the treatment or management of jaundice. The substantiation presented is an indicative of the utilization of the plants in the prevention and/or treatment of jaundice. Though there is tremendous ethnopharmacological information about the plants involved in jaundice treatment, only some of them were used in clinical trials and have been explored for their mechanism of action. These studies will be covered in the next part of the review.

## Author contributions

DT, AM, EP, ZM, YL, and AA have written the first draft of the manuscript. AS, SN, LH, and JH revised and improved the first draft. All authors have seen and agreed on the finally submitted version of the manuscript.

### Conflict of interest statement

The authors declare that the research was conducted in the absence of any commercial or financial relationships that could be construed as a potential conflict of interest.

## Footnotes

^1^Definition of Icterus (2016). Available online at: http://www.medicinenet.com/script/main/art.asp?articlekey=3890 (Accessed on September 18 2016).
